# Molecular characterization and transcription analysis of DNA methyltransferase genes in tomato (*Solanum lycopersicum*)

**DOI:** 10.1590/1678-4685-GMB-2018-0295

**Published:** 2020-03-06

**Authors:** Xuhu Guo, Qian Xie, Baoyuan Li, Huanzhen Su

**Affiliations:** 1 Shanxi Datong University, School of Life Sciences, Datong, China.; 2 Shanxi Datong University, Applied Biotechnology Institute, Datong, China.

**Keywords:** DNA methylation, tissue-specific expression, abiotic stress, fruit ripening, tomato

## Abstract

DNA methylation plays an important role in plant growth and development, gene expression regulation, and maintenance of genome stability. However, only little information regarding stress-related DNA methyltransferases (MTases) genes is available in tomato. Here, we report the analysis of nine tomato MTases, which were categorized into four known subfamilies. Structural analysis suggested their DNA methylase domains are highly conserved, whereas the N-terminals are divergent. Tissue-specific analysis of these MTase genes revealed that *SlCMT2*, *SlCMT3*, and *SlDRM5* were expressed higher in young leaves, while *SlMET1*, *SlCMT4*, *SlDRM7*, and *SlDRM8* were highly expressed in immature green fruit, and their expression declined continuously with further fruit development. In contrast, *SlMETL* was highly expressed in ripening fruit and displayed an up-regulated tendency during fruit development. In addition, the expression of *SlMET1* in the ripening of mutant *rin* and *Nr* tomatoes is significantly higher compared to wild-type tomato, suggesting that *SlMET1* was negatively regulated by the ethylene signal and ripening regulator MADS-RIN. Furthermore, expression analysis under abiotic stresses revealed that these MTase genes were stress-responsive and may function diversely in different stress conditions. Overall, our results provide valuable information for exploring the regulation of tomato fruit ripening and response to abiotic stress through DNA methylation.

## Introduction

DNA methylation plays a crucial role in gene expression regulation, maintenance of genome stability, and it controls the transcription of invading and mobile DNA elements (Law and Jacobsen, 2010; Feng and Jacobsen, 2011). Plants possess four types of DNA methyltransferases (MTases), namely methyltransferase (MET), chromomethylase (CMT), domains rearranged methyltransferase (DRM), and DNA methyltransferase homologue 2 (DNMT2) (Law and Jacobsen, 2010). MET maintains CG methylation of heterochromatic regions enriched with transposable elements (TEs) and repeats, and genic regions (Cokus *et al.*, 2008; Lister *et al.*, 2008). CMT and DRM mediate CHG and CHH (H=A/C/T) methylation (Law and Jacobsen, 2010; Kohler *et al.*, 2012). DNMT2 has a novel transfer RNA (tRNA) methyltransferase activity (Goll *et al.*, 2006; Jeltsch *et al.*, 2006), but its role in C5 DNA methylation remains largely unknown (Pavlopoulou and Kossida, 2007).

DNA methyltransferases genes have been found in many plant species, such as tobacco, rice, *Arabidopsis*, wheat, maize, *Physcomitrella*, and legumes (Dai *et al.*, 2005; Wada, 2005; Pavlopoulou and Kossida, 2007; Fulnecek *et al.*, 2009; Sharma *et al.*, 2009; Malik *et al.*, 2012; Rohini *et al.*, 2014). DNA methylation is primarily catalyzed by the DNA methyltransferase family. DNA methyltransferase plays an important role in plant development, transcriptional regulation, and metabolic pathway control. For example, the triple mutation of *drm1drm2cmt3* leads to delayed growth, small plant size, and partial barrenness in *Arabidopsis* (Cao and Jacobsen, 2002). DNA methylation is also involved in tomato fruit ripening. The Colorless non-ripening (*Cnr*) mutation inhibits normal tomato ripening due to methylation of the *SBP-CNR* gene promoter (Manning *et al.*, 2006; Giovannoni, 2007). Chen *et al.* (2015) recently reported on the role of a chromomethylase (SlCMT3) for the stable methylation of the promoter region of the *Cnr* gene.

Plants are continuously affected by abiotic or biotic environments, and thus have developed notable abilities to regulate their physiological and developmental mechanisms through gene expression regulation in response to these environmental perturbations (Zhou *et al.*, 2007). Epigenetic mechanisms, including DNA methylation and histone modification, play important roles in regulating gene expression in plant responses to environmental stress (Razin and Cedar, 1992; Cullis, 2005; Boyko *et al.*, 2007; Boyko and Kovalchuk, 2008). For instance, salinity and water stress can trigger demethylation at coding regions of certain genes and subsequently initiate their expression (Choi and Sano, 2007). To the contrary, satellite sequences can be hypermethylated, especially in CHG sequences after salt stress (Dyachenko *et al.*, 2006). Low-temperature stress reduces the amount of methyltransferase in corn (*Zea mays L.*) (Steward *et al.*, 2000).

In this study, based on the complete sequence of tomato genomes, as well as expression profiles at different tissues/stages and abiotic stresses (low temperature and salt), the nine tomato MTases were analyzed and characterized through an approach combining bioinformatics and expression experiments. Our study provides valuable information for functional research of DNA methyltransferase genes in tomato.

## Materials and Methods

### DNA and protein sequence analysis

The protein sequences of *Arabidopsis* and rice MTases (Table S1) were used to search for the amino acid sequences of tomato MTases in the NCBI (http://blast.ncbi.nlm.nih.gov/Blast.cgi) and Sol Genomics Network (SGN) (http://solgenomics.net/) databases using the Blastp tool with the filter-off option and a cut-off value of 1 e^-10^. The genomic DNA sequences of these nine genes were obtained from the SGN. In order to analyze the exons and introns of genomic DNA, sequence alignment between CDS (coding sequence) and genomic DNA was done by MultAlin. The gene structures of the DNA MTases in tomato were generated using the GSDS. Molecular weight (Mw), isoelectric points, and grand average of hydropathicity (GRAVY) were estimated with the ExPASy compute Mw tool. Conserved structure domains were annotated based on ScanProsite and the Pfam protein family database. Motif detection was dependent on MEME (Timothy *et al.*, 1994). The phylogenetic tree was constructed using MEGA 5.02 software and the neighbor-joining method with the following parameters: bootstrap analysis of 1,000 replicates, Poisson model, and pairwise deletion. The numbers at the nodes indicate the bootstrap values. Promoter element analysis was performed using plant CARE and PLACE, which is a database of motifs found in plant *cis-*acting regulatory DNA elements.

### Plant material

Tomato (*Solanum lycopersicum* Mill. cv. Ailsa Craig) seedlings were grown under greenhouse conditions (16 h days at 27 °C and 8 h nights at 19 °C). For organ-specific expression profiling of genes, tomato roots, stems, leaves, sepals, flowers and fruit pericarp tissues of different periods were harvested. Roots and stems were collected from 45-day-old tomato seedlings based on their uniformity. The leaves were taken from three different parts of 65-day-old tomato plants, namely young leaves (3 leaves of new growth), mature leaves (5 to 7 leaves from top to bottom) and senescent leaves (8 to 10 leaves from top to bottom). Sepals and petals were collected at the same time. Flowers were marked at anthesis and fruit development was recorded as days post-anthesis (DPA). Fruits ripening was divided into five stages, namely IMG (immature green, 28 DPA), MG (mature green, 35 DPA, full fruit expansion but no obvious color change), B (breaker, fruit showing the first signs of ripening-associated color change from green to yellow), B4 (4 days after breaker) and B7 (7 days after breaker).

### Expression analysis of DNA MTase genes by gene microarray

Microarray expression data were obtained from the tomato Gene Chip platform of Genevestigator (https://www.genevestigator.com/gv/). The nucleotide sequences of DNA MTase genes were used as query sequences to blast against all of the gene probe sequences from the Affymetrix Gene Chip (http://www.affymetrix.com/), and the best homologous probes were selected and used to carry out search in the Affymetrix Tomato Genome Array platform.

### Stress treatments

Potted 35-day-old tomato seedlings chosen based on their uniformity were used for all stress treatments. For salt stress treatment, the roots of tomato seedlings were submerged in a solution containing 250 mM NaCl for 0, 1, 2, 4, 8, 12, and 24 hours, and the young leaves of the treated seedlings and controls were collected. For low temperature stress treatment, the whole potted tomato seedlings were incubated at 4 °C for 0, 1, 2, 4, 8, 12, and 24 hours, after which the leaves were collected (Zhu *et al.*, 2014). All stress treatments were performed with three biological replicates.

### RNA isolation and quantitative RT-PCR analysis

Total RNA was extracted from tomato tissues with the Trizol reagent (Invitrogen, Shanghai, China). Genomic DNA pollution was eliminated with DNase I (Promega, Beijing, China) in the presence of RNase inhibitor (Takara Biotechnology, Japan). Poly (A)+RNA was used as a template for synthesis of first-strand cDNA. Complementary DNA was synthesized by M-MLV reverse transcriptase (Promega, Beijing, China) at 37 °C for 1 h. The quantitative RT-PCR reaction system and conditions were performed as in our previous report (Guo *et al.*, 2016). The tomato *CAC* and *EF1*α genes were used as internal controls under normal growth conditions (Expósito-Rodríguez *et al.*, 2008) and abiotic stress (Nicot *et al.*, 2005), respectively. The analysis of gene relative expression levels was conducted using the 2^-DDCT^ method (Livak and Schmittgen, 2001). All primers used for quantitative RT-PCR are listed in Supplementary Table S2. The mean values of three independent experiments were calculated, and the standard deviations (± SD) were indicated.

### Statistical analysis

All experiments were conducted with three biological replicates. Statistical data were analyzed by Origin 8.0 software, and performed using the Student’s *t*-test (SPSS 22.0). Values of *p* < 0.05 were considered significant. Data are presented as mean ± SD.

## Results

### Identification of tomato DNA MTases and sequence analysis

**Figure 1:**
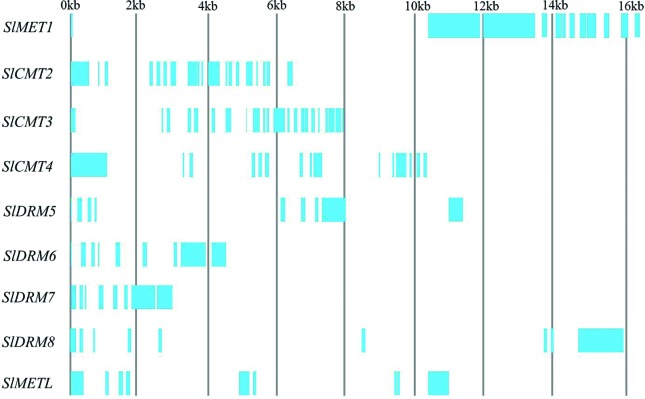
Gene structure of methyltransferases (MTases) in tomato. Intron-exon organization is shown in the upper panel. Exons are shown as blue boxes and introns are represented by spaces between the blue boxes.

Firstly, the data for 11 and 10 MTases in *Arabidopsis* and rice (Table S1) was collected from NCBI, respectively. Based on these data, nine MTases were identified in tomato through Blastp (Table 1). The open reading frame (ORF) length of these genes varies from 1.1 to 4.6 kb, and their protein length ranged from 381 to 1559 amino acids. All the deduced polypeptides are hydrophilic. In addition, Figure 1 shows the intron-exon organization (number of introns and exons) of nine MTases in tomato. The coding regions of CMT subfamily genes are interrupted by 14-21 introns (Figure 1). MET gene (*SlMET1*) length is approximately 4.6 kb in tomato, harboring 12 exons. The length of the DRM subfamily genes in tomato varies from 1.8-2.1 kb with nine exons. DNMT2 gene (*SlMETL*) is smallest in length (1.1 kb) harboring nine exons. Genomic distribution of these tomato MTase genes was also analyzed. Nine tomato MTases genes are dispersedly located on chromosomes, with one MTase variant mostly located on a single chromosome (Table 1), suggesting at least partial influence of WGD in the diversification of the MTases family in tomato, rather than gene duplication.

**Table 1:** Overview of MTases genes identified in tomato.

Gene name	ORF length^a^ (bp)	Deduced polypeptide^b^				Chromosome number	Accession number^c^
		Length (aa)	Mol wt.(kDt)	PI	GRAVY		
*SlMET1*	4680	1559	175.03	6.03	-0.517	ch11 18811587-18827974	AJ002140
*SlCMT2*	2802	933	104.50	5.40	-0.581	ch12 65430879-65437290	XM_004252792
*SlCMT3*	2235	808	91.17	4.90	-0.376	ch01 756827-764851	XM_004228549
*SlCMT4*	2667	888	100.04	8.82	-0.655	ch08:292101-303500	XR_182971
*SlDRM5*	1812	603	68.03	4.79	-0.504	ch02 29084337-29096121	EU344815
*SlDRM6*	1830	609	69.09	5.16	-0.464	ch10 59372041-59376567	SGN-U321564
*SlDRM7*	1824	607	68.71	4.75	-0.492	ch04 185839-189158	TC161581
*SlDRM8*	2100	699	78.82	5.45	-0.411	ch05:62542201-62559200	SGN-U325992
*SlMETL*	1146	381	43.42	5.44	-0.312	ch08 53192484-53203494	XP_004245195

a Length of open reading frame in base pairs.

b Length of amino acids, molecular weight (kDa), isoelectric point (pI), and grand average of hydropathicity (GRAVY) of the deduced polypeptide.

c GenBank, SGN or TIGR accession number of tomato MTases genes.

### Conserved domains and phylogenetic analysis

**Figure 2:**
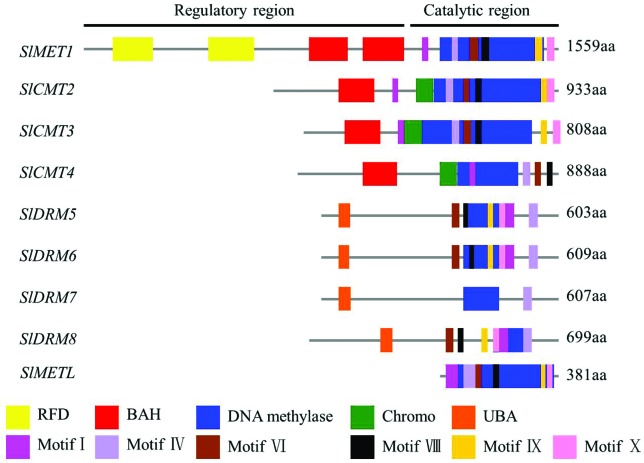
Protein structure of methyltransferases (MTases) in tomato. The domain and motif organization are shown in the upper panel. Different domains and motifs are shown in different colors along with the consensus sequence of the predicted motifs as indicated in the legend.

Alignment of the amino acid sequences of these nine tomato DNA MTases revealed that tomato MTases genes possess a regulatory region and a catalytic region with conserved motifs that are arranged in a specific order. Six highly conserved motifs I, IV, VI, VIII, IX, and X were identified in the methyltransferase domain via MEME analysis in the nine MTases (Figure 2). We found that each subfamily of tomato MTase has a characteristic arrangement of these motifs in the catalytic region. MET members showed the order of motifs as I, IV, VI, VIII, IX, and X. In CMT members, chromodomain was present between the conserved motifs I and IV with the rest of the arrangement similar to the MET members. It is interesting to note that *SlCMT4* appeared to lack the IX and X domains. DRM members showed the order of motifs as VI, VIII, IX, X, I, and IV except in *SlDRM7*, which only possesses the IV motif. Only one ubiquitin-associated domain (UBA) was present in the DRM family members. Similar to MET, DNMT2 member showed the order of motifs as I, IV, VI, VIII, IX, and X, but no regulatory region (Figure 2).

**Figure 3:**
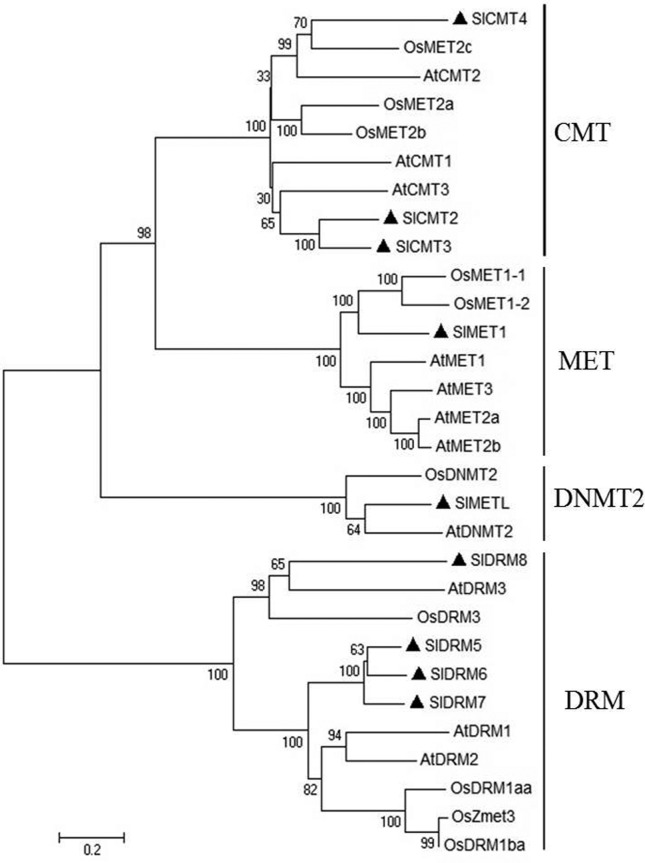
Phylogenetic tree of methyltransferases (MTases) domain protein sequences in plants. Tomato MTases genes are marked with black triangles. Accession numbers for other proteins are listed in Table S1. Os - *Oryza sativa*, At – *Arabidopsis*.

MTases, including replication foci domain (RFD), bromo adjacent homology (BAH), and methyltransferase domains were classified as MET subfamily members, whereas members with the Chr domain, along with BAH, and methyltransferase domain were placed in the CMT subfamily (Figure 2). Members harboring both UBA and methyltransferase domains were grouped into a DRM subfamily (Figure 2). DNMT2 subfamily members seem to lack any amino-terminal regulatory domain and include only a methyltransferase domain (Figure 2). In tomato, a total of three MTase genes were identified as CMT, one as MET, four as DRM, and one as DNMT2 members (Figure 3); in *Arabidopsis*, three members belonged to CMT (AtCMT1, 2, and 3), four to MET (AtMET1, AtMET2a, AtMET2b, and AtMET3), three to DRM (AtDRM1, 2, and 3) and one to DNMT2 (AtDNMT2) families. Similarly, there were three CMTs (OsMET2a, OsMET2b, and OsMET2c), two METs (OsMET1-1 and OsMET1-2), four DRMs (OsDRM1aa, OsDRM1ba, OsDRM3, and OsZmet3) and one DNMT2 (OsDNMT2) in rice (Sharma *et al.*, 2009). As shown in Figure 3, four clades (CMT, MET, DNMT2, and DRM) were clearly distinguished with support values close to 100. The CMT subfamily contained nine proteins, among which were three tomato proteins (SlCMT2, SlCMT3, and SlCMT4). The clades MET and DNMT2 included only SlMET and SlMETL, respectively. The DRM clade contained four tomato proteins (SlDRM5, SlDRM6, SlDRM7, and SlDRM8). Thus, our evolutionary analysis results showed good consistency with the classification results.

### Transcription pattern of DNA MTase genes in wild-type tomato and mutants

**Figure 4:**
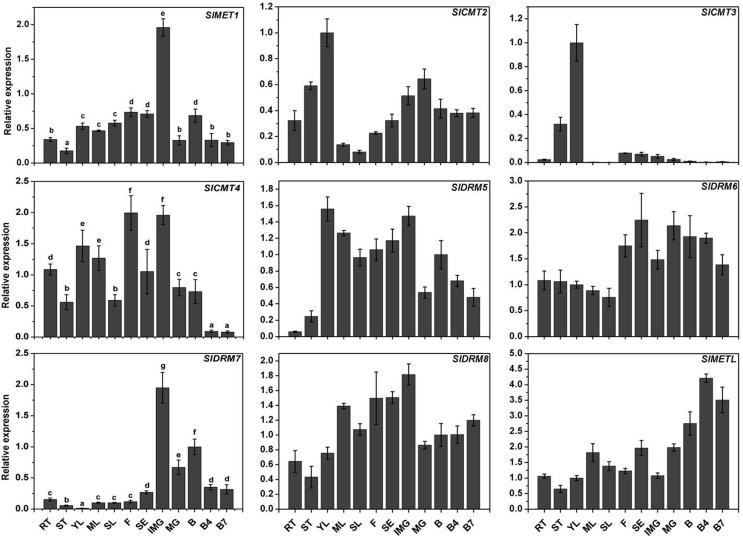
Expression profiles of MTase genes in different tissues and different developmental stages in wild-type tomato. RT, root; ST, stem; YL, young leaf; ML, mature leaf; SL, senescent leaf; F, flower; SE, sepal; IMG, immature green; MG, mature green; B, breaker; B4, 4 days after breaker stage; B7, 7 days after breaker stage. Data are reported as mean ± SD of three independent experiments. Significant differences (p < 0.05) are denoted by different letters.

**Figure 5:**
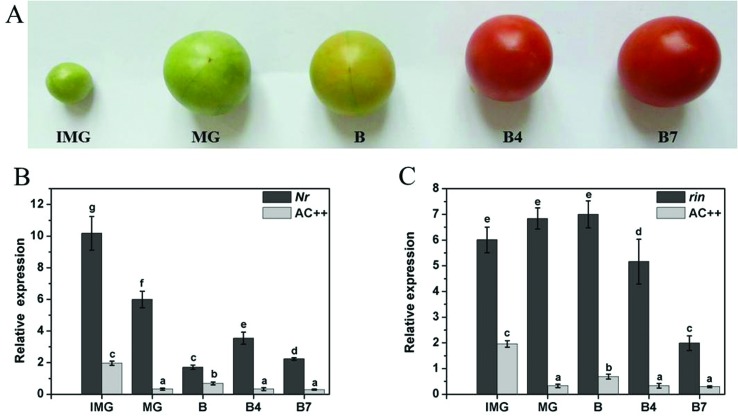
Expression profiles of SlMET1 in different fruit developmental stages in wild-type tomato AC++ (A) and mutant tomato Nr (B)/rin (C). IMG, immature green; MG mature green; B breaker; B4, 4 days after breaker stage; B7, 7 days after breaker stage. Data are reported as mean ± SD of three independent experiments. Significant differences (p < 0.05) are denoted by different letters.

To elucidate the tissue/organ expression patterns of MTase genes in tomato, quantitative RT-PCR was carried out using cDNAs from different tissues and development stages. Figure 4 shows that *SlCMT2* was highly expressed in young leaves, mature green fruits, and stems, while its expression was down-regulated continuously during leaf development. *SlCMT3* was also predominantly expressed in young leaves and its transcription level declined continuously with further fruit ripening. *SlCMT4* was highly expressed in flowers and immature green fruits relative to other tissues, while its expression was down-regulated continuously during fruit development. The expression pattern of *SlMET1* was very similar to that of *SlDRM7*. Their transcripts both reached a maximum level in immature green fruits. *SlDRM5* was highly expressed in young leaves. During fruit development, *SlDRM5* transcripts reached a maximum in immature green fruit and then decreased. Interestingly, the expression of *SlDRM6* in the reproductive stage was higher than in the vegetative growth stage. *SlDRM8* expression was slightly higher in flowers, sepals, and immature green fruits than in other tissues. *SlMETL* expression was higher in ripening fruits and displayed an up-regulated tendency during fruit development. Spatial and temporal expression of *SlMET1*, *SlCMT2*, *SlDRM5*, *SlDRM7*, *SlDRM8*, and *SlMETL* were basically consistent with microarray expression data (Figure S1). Besides, it is worthy of note that the expression level of *SlMET1* in the tomato ripening mutants *rin* and *Nr* was significantly higher compared to wild-type tomato (Figure 5).

### Tomato DNA MTases are involved in abiotic stress response

**Figure 6:**
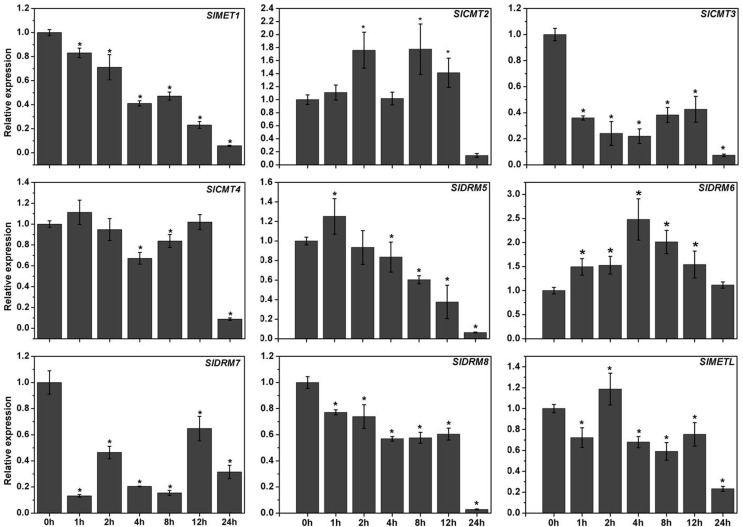
Quantitative RT-PCR analysis of the MTase genes under low temperature stress. The relative expression levels were normalized to 1 in unstressed plants (0 h). Data are reported as mean ± SD of three independent experiments. The asterisks indicate statistically significant differences between the treated and unstressed seedlings (p < 0.05).

To further study the potential functions of these tomato DNA MTases genes, we carried out expression analyses under low temperature and salt stress conditions by quantitative RT-PCR. For low-temperature treatment (Figure 6), we noted that the expression of *SlMET1* and *SlDRM5* was inhibited by low temperature and decreased gradually. The transcript levels of *SlCMT3*, *SlCMT4*, *SlDRM7*, *SlDRM8*, and *SlMETL* were also decreased under low temperature stress, especially *SlCMT3* and *SlDRM7*, which were sharply down-regulated at 1 h. Additionally, *SlCMT2* and *SlDRM6* were up-regulated slightly during the first 12 hours of treatment, but a significant decrease in *SlCMT2* mRNA was detected at 24 h.

**Figure 7:**
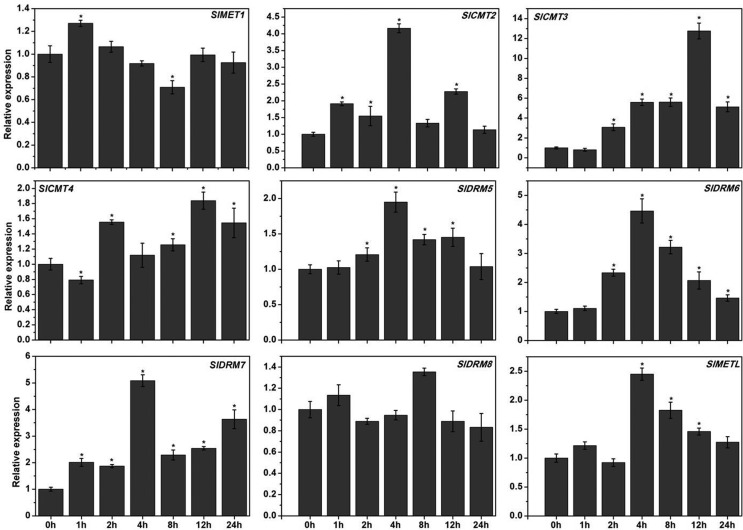
Quantitative RT-PCR analysis of the MTase genes in young leaves under NaCl stress. Tomato seedlings were grown with 250 mM NaCl. The relative expression levels were normalized to 1 in unstressed leaves (0 h). Data are reported as mean ± SD of three independent experiments. The asterisks indicate statistically significant differences between the treated and unstressed seedlings (p < 0.05).

For salt treatment (Figure 7), the induction of *SlCMT2* gene expression was observed; it peaked at 4 h and returned to basal level at 24 h. The expression of *SlCMT3* in leaves was significantly up-regulated at 12 h by about 13-fold. *SlCMT4* was slightly down-regulated at 1 h and up-regulated subsequently in leaves. *SlDRM5* and *SlMETL* were induced, and their transcripts peaked at 4 h in leaves. The expression of *SlDRM6* was increased gradually and peaked at 4 h in leaves, with an expression pattern similar to that of *SlDRM7*. Comparatively, the transcript levels of *SlMET1* and *SlDRM8* were less affected in leaves. The above results suggest that these MTases genes may be involved in the response to salt stress.

## Discussion

DNA methylation is an important epigenetic modification established by DNA methyltransferase. Although tomato is a model plant for studying fleshy fruit development and ripening, little is known regarding a comprehensive analysis of MTases in tomato. In the present study, we analyzed tomato MTases and identified three members of CMT, one MET, four DRMs, and one DNMT2 in tomato. Each of the tomato MTases genes has a homologous gene in *Arabidopsis*, suggesting that MTases in tomato might have similar roles as in *Arabidopsis*. In addition, the systematic expression pattern of tomato MTases in different tissues/development stages and abiotic stress provides evidence for diverse functions in various aspects of plant development and abiotic stress responses.

The structural analysis suggested that catalytic DNA methylase domains are highly conserved, whereas the N-terminus, which is regarded as a regulatory region, is divergent (Figure 2). Thus, these nine tomato MTase genes may play different roles in regulating tomato growth and development. MET subfamily members are very similar to the mammalian DNMT1 class (Law and Jacobsen, 2010). Our structural analysis of tomato CMTs (SlCMT2, SlCMT3, and SlCMT4) suggested that the N-terminus of CMT harbors the BAH and Chr domains, which could possibly enhance the binding attraction of CMTs to methylated histones, similar to *Zea mays* CMT3 (Du *et al.*, 2012). Four DRM members were identified in tomato. The N-terminus of DRM possesses the UBA domain, where sequence motifs occur that are usually involved in ubiquitin-mediated proteolysis and contributing to ubiquitin (Ub) binding or ubiquitin-like (UbL) domain binding. Recent findings have established DNMT2 as a tRNA methyltransferase that plays an important function under stress conditions (Schaefer and Lyko, 2010; Thiagarajan *et al.*, 2011). We also investigated one member (*SlMETL*) of the DNMT2 family in tomato, lacking a conserved N-terminal regulatory domain, but possessing a catalytic C-terminal domain, which seems to be characteristic for all DNMT2s.

So far, the characteristics and functions of MTases in *Arabidopsis* have been studied clearly (Finnegan and Dennis 1993), but there is very little knowledge of their expression profiles in different tissues/developmental stages in tomato (Teyssier *et al.*, 2008). In this study, we investigated the expression pattern of the nine DNA MTases genes in different tissues/stages (Figure 4), suggesting overlapping and specific roles during tomato development. The higher expression of *SlMET1* in IMG fruits in tomato suggested its role in the maintenance of methylation in early stages of fruit development. This is different from the expression of MET members in *Arabidopsis* and rice, which was higher in the early stages of flower and seed development (Saze *et al.*, 2003; Xiao *et al.*, 2003; Kinoshita *et al.*, 2004; Sharma *et al.*, 2009; Schmidt *et al.*, 2013). The ANAERO2CONSENSUS and CANBNNAPA elements (Ellerström *et al.*, 1996) regulating fruit and embryo development respectively, were identified in the promoter of *SlMET1* (Table S3), suggesting *SlMET1* might be related with fruit development, which was confirmed by its high expression in fruit. *SlCMT4* was highly expressed in flower, immature green fruit, and young leaf, which was coincident with a previous report (Teyssier *et al.*, 2008). *SlMETL* showed the highest expression in B4 fruits, and *SlDRM6* expression in reproductive stage was significantly higher than in vegetative growth stage, suggesting that these proteins may play an important role in tomato reproductive stage. Interestingly, *SlCMT3* was specifically expressed in young leaves, suggesting that *SlCMT3* may play critical roles in tomato leaf development. Consistent with its function in the DNA methylation maintenance, the tomato CMT was predominantly expressed in actively replicating cells in young leaves and roots. Additionally, it is noteworthy that *SlMET1* and *SlDRM7* were specifically expressed in immature green fruit, suggesting their useful application in fruit ripening and development.

Epigenetic modifications play an important role in response to environmental stimuli (Chinnusamy and Zhu, 2009; Gutzat and Mittelsten, 2012). For example, most of the MTases genes in pigeon pea are responsive to NaCl and extreme temperature (Rohini *et al.*, 2014). To further study the potential functions of the nine tomato MTases genes, we examined their expression under various stress conditions by quantitative RT-PCR. We found that most of the DNA MTases genes in tomato are responsive to stress treatments, including NaCl and low temperature (Figures 6 and 7), and the differential expression profiles indicated that they may function diversely in different stress conditions. Although *SlDRM5* and *SlDRM6* appeared highly similar in protein structure (Figure 2) and transcription in native leaves (Figure 4), the transcriptional responses to salt stress were remarkably different, being increased by about 2 times for *SlDRM5* and 4.5 times for *SlDRM6* after 4 h of treatment (Figure 7). This probably correlates with number of GAAAAA (GT1GMSCAM4) promoter *cis-*elements, known to be responsible in wound repair (Table S3).

DNA methylation is involved widely in the regulation of the temporal and spatial gene expression in plants. DNA methyltransferase inhibitor 5-azacytidine induces tomato fruit premature ripening (Zhong *et al.*, 2013), and it is demonstrated that DNA methylation contributes to the regulation of fruit ripening. In this study, we observed that *SlMET1* was highly expressed in immature green fruit and then declined during fruit ripening, which was consistent with a previous report by Teyssier *et al.* (2008). Interestingly, the expression levels of *SlMET1* in the tomato ripening mutants *rin* and *Nr* are higher than in wild type tomato (Figure 5), suggesting that *SlMET1* is negatively regulated by the ethylene signal and ripening-related transcriptional factor MADS-RIN. We speculate that the abnormal fruit ripening in the mutants *Nr* and *rin* might be related to the concurrent hypermethylation of multiple ripening-related genes by DNA methyltransferase SlMET1.

In summary, based on bioinformatics and transcriptional pattern analysis, the nine MTase genes identified in tomato could be involved in tomato development and abiotic stress responses. This study also provided valuable information about tomato MTase genes associated with fruit ripening.

## Supplementary material

The following online material is available for this article:

Table S1 - Basic information of DNA methyltransferases from *Arabidopsis* and rice.

Table S2 - Primer pairs used in quantitative RT-PCR analysis.

Table S3 - Putative *cis-*elements enriched in the promoters of tomato MTases genes.

Figure S1 - Microarray expression data.
