# Supplementary material for: Molecular characterization and transcription analysis of DNA methyltransferase genes in tomato (Solanum lycopersicum)
Source: Genet Mol Biol. 2020 Mar 6;43(1):e20180295. doi: 10.1590/1678-4685-GMB-2018-0295 (PMC7197986; doi:10.1590/1678-4685-GMB-2018-0295)
Supplement: Supplementary file 1 [file 1415-4757-GMB-43-1-e20180295-s1.pdf]

Supplementary Material to "Molecular characterization and transcription analysis of DNA methyltransferase genes in tomato (*Solanum lycopersicum*)"

Table S1 - Basic information of DNA methyltransferases from *Arabidopsis* and rice.

Protein name	Accession number	Gene loci	Protein length (aa)
AtMET1	NP_199727	At5g49160	1534
AtMET2a	NP_193150	At4g14140	1519
AtMET2b	NP_192638	At4g08990	1512
AtMET3	NP_193097	At4g13610	1404
AtDRM1	NP_197042	At5g15380	624
AtDRM2	NP_196966	At5g14620	626
AtDRM3	NP_566573	At3g17310	710
AtCMT1	NP_565245	At1g80740	791
AtCMT2	NP_193637	At4g19020	1295
AtCMT3	NP_177135	At1g69770	839
AtDNMT2	NP_568474	At5g25480	383
OsMET1-1	XP_015628331	LOC4334435	1527
OsMET1-2	BAT00336	Os07g0182900	1497
OsMET2a	XP_015613201	LOC4347954	907
OsMET2b	XP_015630016	LOC4332128	1059
OsMET2c	XP_015639449	LOC4338140	1319
OsDRM1aa	ABA91139	LOC_Os11g01810	473
OsDRM1ba	XP_015630994	LOC4331357	675
OsDRM3	XP_015639540	LOC4337721	680
OsDNMT2	XP_015621754	LOC9268926	374
OsZmet3	AAN61474	OSJNBb0043C10.1	881
