# Supplementary material for: Molecular characterization and transcription analysis of DNA methyltransferase genes in tomato (Solanum lycopersicum)
Source: Genet Mol Biol. 2020 Mar 6;43(1):e20180295. doi: 10.1590/1678-4685-GMB-2018-0295 (PMC7197986; doi:10.1590/1678-4685-GMB-2018-0295)
Supplement: Supplementary file 2 [file 1415-4757-GMB-43-1-e20180295-s2.pdf]

Supplementary Material to "Molecular characterization and transcription analysis of DNA methyltransferase genes in tomato (*Solanum lycopersicum*)"

Table S2 - Primer pairs used in quantitative RT-PCR analysis.

Prime code	Primer sequences (5' →3')	Products length (bp)
SlCAC-Q-F	CCTCCGTTGTGATGTAACTGG	173
SlCAC-Q-R	ATTGGTGGAAAGTAACATCATCG	
SlEF1α-Q-F	TACTGGTGGTTTTGAAGCTG	150
SlEF1α-Q-R	AACTTCCTTCACGATTTCATCATA	
SlMET1-Q-F	GGGTTGTCCGAGCGATGA	250
SlMET1-Q-R	GCTTTTGGGCCAATACGTAGA	
SlCMT2-Q-F	AAGTAGATGGAATGGAGCTAGGG	256
SlCMT2-Q-R	ACATCTGCTCGACTAAAATGGC	
SlCMT3-Q-F	AGCATTGGCATTGAAAGGATT	255
SlCMT3-Q-R	CCATTGTCCTAAATTAGCAACTAACA	
SlCMT4-Q-F	CTTGGCACAAAACTCTCTGGTC	125
SlCMT4-Q-R	TTCTCAACACCTTCATTCCTAACAT	
SlDRM5-Q-F	TGTGGTCCAGAAGCATCGG	264
SlDRM5-Q-R	TCTTCGGACAATTGCAGAAACT	
SlDRM6-Q-F	TTGATTATGTTCGGATATTGGATG	238
SlDRM6-Q-R	GATACCTGGGTTCTGGAAAAACT	
SlDRM7-Q-F	AACAGGGTGACTAGAGACGGACT	225
SlDRM7-Q-R	GATGGCACTAAGAGAGTTACTTTGTG	
SlDRM8-Q-F	GTTTTATGCTTTTGCTGTGGC	196
SlDRM8-Q-R	TGTCCCCCATAGGCATTGTA	
SlMETL-Q-F	TTGGGCTGTGTATGTGTTTGG	260
SlMETL-Q-R	CAAGCCACCAATTCCACTGTA	
