# Supplementary material for: Molecular characterization and transcription analysis of DNA methyltransferase genes in tomato (Solanum lycopersicum)
Source: Genet Mol Biol. 2020 Mar 6;43(1):e20180295. doi: 10.1590/1678-4685-GMB-2018-0295 (PMC7197986; doi:10.1590/1678-4685-GMB-2018-0295)
Supplement: Supplementary file 3 [file 1415-4757-GMB-43-1-e20180295-s4.pdf]

Supplementary Material to "Molecular characterization and transcription analysis of DNA methyltransferase genes in tomato (*Solanum lycopersicum*)"

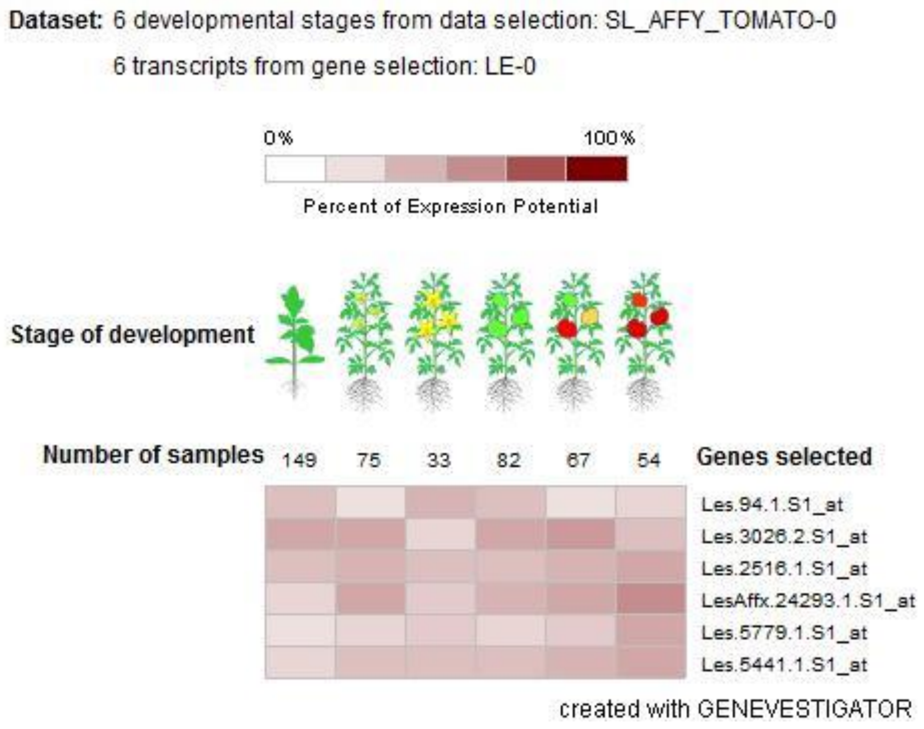

Figure S1 - Microarray expression data obtained using Genevestigator (https://www.genevestigator.com/gv/) with the tomato Gene Chip platform. SlMET1, Les.94.1.S1_at; SlCMT2, Les.3026.2.S1_at; SlDRM5, Les.2516.1.S1_at; SlDRM7, LesAffx.24293.1.S1_at; SlDRM8, Les.5779.1.S1_at; SlMETL, Les.5441.1.S1_at.
