# Supplementary material for: Molecular characterization and transcription analysis of DNA methyltransferase genes in tomato (Solanum lycopersicum)
Source: Genet Mol Biol. 2020 Mar 6;43(1):e20180295. doi: 10.1590/1678-4685-GMB-2018-0295 (PMC7197986; doi:10.1590/1678-4685-GMB-2018-0295)
Supplement: Supplementary file 4 [file 1415-4757-GMB-43-1-e20180295-s3.pdf]

Supplementary Material to "Molecular characterization and transcription analysis of DNA methyltransferase genes in tomato (*Solanum lycopersicum*)"

Table S3 - Putative *cis*-elements enriched in the promoters of tomato MTases genes.

Factor or Site Name	Signal Sequence	*SlMET1*	*SlCMT2*	*SlCMT3*	*SlCMT4*	*SlDRM5*	*SlDRM6*	*SlDRM7*	*SlDRM8*	*SlMETL*	Function
MYB1AT	WAACCA	1	4	2	0	0	2	1	0	1	dehydration
MYB2CONSENSUSAT	YAACKG	3	3	2	2	1	0	0	3	0	dehydration
GT1GMSCAM4	GAAAAA	1	1	3	2	3	6	3	0	0	Salt and pathogen
WBOXNTERF3	TGACY	5	3	5	5	0	0	2	3	3	Wounding
OSE2ROOTNODULE	CTCTT	7	3	4	1	4	6	1	3	3	Wounding
ACGT element	ACGT	4	3	9	1	1	3	4	1	2	dehydration and etiolation
MYCCONSENSUSAT	CANNTG	11	10	5	4	9	12	10	5	4	Low temperature
ANAERO2CONSENSUS	AGCAGC	8	1	0	0	0	0	0	0	0	fruit
CANBNNAPA	CNAACAC	3	1	0	0	1	2	1	2	1	embryo
ERELEE4	AWTTCAAA	1	1	0	0	1	1	0	2	0	Ethylene

N=A/T/G/C; W=A/T; Y=T/C.
